# Critical Psychiatry Debates; A Neglected Tool in Undergraduate Medical Education

**DOI:** 10.15694/mep.2021.000055.1

**Published:** 2021-02-25

**Authors:** Ahmad Allam, Ashvin Kuri, Ali Ajaz, Saman Ahmed, Peter Carter, Ania Korszun

**Affiliations:** 1 North East London Foundation Trust; 2 Barts & The London Medical School; 3 Centre for Psychiatry

**Keywords:** Debates, Psychiatry, Students, Healthcare, Education, Skills, Attitudes, Critical, Communication, Conflict.

## Abstract

This article was migrated. The article was marked as recommended.

**Background:** Healthcare professionals must adapt to everyday clinical controversies using their critical thinking and communication skills. Educational debates nurture these skills producing a well-rounded clinician. Their value is well established in healthcare education, yet They are not commonly employed, and there is no literature on their use in undergraduate Psychiatry teaching.

**Methods:** We planned and implemented a one-off educational debate lesson as part of the teaching program of fourth-year medical students on their Psychiatry clinical placements. We collected and analyzed the students’ feedback.

**Results:** Although the majority of students had no experience of debates, 79% found the learning event positive. The students also reported improvement in their confidence (58%), oral presentation skills (37%), critical thinking skills (71%) and the ability to cope with conflict (54%). There was a positive shift in their attitude towards Psychiatry (71%) and the chances of choosing it as a future speciality (33%).

**Conclusion:** Our results showcase the benefit of using debates in shaping future doctors’ non-clinical skills and practice attitudes. In this paper, we discuss a thematic analysis of students’ feedback comments and reflect on several points in the planning and delivery of educational debates. We also include practical recommendations for future applications.

## Introduction

With motivation and appropriate skills, health care professionals can learn to address the controversies of clinical practice, and through educational debates, we can nurture such skills ( Ganguli and Rancurello, 1990). Snider and Schnurer (2006, p. 6) defined debates as “structured communications on topics of interest”. They facilitate adult learning via constructivist ( Ang *et al.*, 2019), social ( Kennedy, 2007) and transformative learning theories ( Kedraka and Kourkoutas, 2018). They are an active learning process focusing the educational event on the learners ( Ang *et al.,* 2019), and their educational value is well documented in learners’ perceptions ( Bonwell and Eison, 1991 p. 45-46), educators’ observations ( Bonwell, 1996) and learning outcomes ( Malone and Michael, 2018).

Educational debates improve communication skills ( Doody and Condon, 2012), the ability to think critically ( Doody and Condon, 2012), attitudes to complex fields ( Malone and Michael, 2018), motivation ( Hartin *et al.*, 2017), leadership ( Hartin *et al.*, 2017), and general academic performance ( Malone and Michael, 2018). They focus on high-level intellectual processes; Analysis, Evaluation, and Creation, thus achieving a more advanced level of knowledge ( Scannapieco, 1997). Educators find debates more rewarding, exciting and beneficial compared to orthodox lectures ( Bonwell, 1996; Musselman, 2004). Moreover, compared with standard methods of active learning, such as problem-based learning or group discussions, debates are considered less exhaustive of faculty resources ( Omelicheva, 2006), and their structure gives an equal chance for all learners to participate ( Dundes, 2001).

Debates have been applied in many fields of healthcare education from ethics ( *Hanna et al.*, 2014) to dentistry ( Darby, 2007) and pediatric surgery ( Ong and Narasimhan, 2010). Their uses are not restricted to esoteric or controversial topics as they have been successfully extended to case-based discussions ( Ong and Narasimhan, 2010) and journal clubs ( Toor, Samai and Wargo, 2017). In Psychiatry, debates have been used in postgraduate teaching ( Ganguli and Rancurello, 1990). Although Keynejad *et al.* (2017) involved medical students in their national psychiatry debate event, they did not focus on using debate in undergraduate teaching.

In this paper, we share our experience of using debates in undergraduate Psychiatry teaching, outlining the structure used, students’ perceptions, and instructors’ reflections, including recommendations for future application.

## Methods

We designed this activity to complement the experiential learning for fourth-year medical students at Barts and The London School of Medicine on their five-week clinical Psychiatry placements. We aimed to improve the students’ communication and critical thinking skills and positively influence their attitudes towards Psychiatry.

A focus group of four students expressed enthusiasm for using debates as a novel learning method. Their opinions provided us with these guiding principles:

• Announcing the activity early with a detailed description.
• Providing an introduction to debates.
• Reducing workload by providing the supporting evidence.
• Reducing performance-anxiety via formative self-assessed outcomes.
• Choosing exciting debate topics.
• Elective participation.

Restricted by resources, we offered this event as a single 120 minutes session opting for a classic structure that allows for greater flexibility ( Berdine, 1984). Table (1) shows the included phases of each debate:

**Table 1:** Phases of each debate motion

Phase	Duration
Transition	Two minutes
Pre-debate vote	One minute
Initial arguments	Five minutes each
Rebuttals	Two & half minutes each
Post-debate vote	One minute
Closing remarks	Two minutes

We defined the participants’ roles to guarantee maximum engagement and learning:

• *The Debaters* would be two individuals or teams of two in which case the allocated time will be split.
• *The Audience* would anonymously vote on each motion before and after the debate. They can also pose questions or comments during the rebuttal phases.
• *The Facilitator* would safeguard the physical and emotional environments by stopping any form of aggression, keep the above structure, and manage the voting polls. Also, they conduct the feedback process and may make a closing remark.

We offered a choice of six motions that met Huber’s (1964) ideal debate criteria;

• Debatable.
• Clearly phrased.
• Attractive to the students.
• Relevant to the course.
• Supported by evidence.
• Achievable in the allocated time.

Resources supporting each argument were collated in an online bank to share. We also provided a 30 minutes introductory session during the students’ induction day to their clinical placements.

## Results

24 out of 26 students participated over three placements in the academic year 2019/2020. Two students opted-out of attending the activity due to other engagements. All 24 students gave feedback using an eleven-item Likert-based form to express agreement or disagreement. Comments boxes under each item allowed them to expand on their responses. This form is in Supplementary File 1. The quantitative results are shown in Table (2) below:

**Table 2:** Quantitative Results

	Much Worse or Very Negative	A Bit Worse or Negative	No Difference or Neutral	A Bit Better or Positive	Improved A Lot or Very Positive
The Learning Experience	0%	0%	21%	54%	25%
Confidence Level	0%	8%	33%	50%	8%
Ability to Cope with Conflict	0%	0%	46%	50%	4%
Presentation Style	0%	0%	63%	33%	4%
Critical Thinking Ability	0%	0%	29%	54%	17%
Attitude towards Psychiatry	0%	0%	29%	42%	29%
Choosing Psychiatry	0%	4%	63%	29%	4%

Also, 63% of the students reported no prior experience of debating and yet 70% recommended having more debates in their MBBS curriculum. As described above, the feedback/self-assessment tool invited the students to write comments under each question to help us capture their experience more accurately. The qualitative feedback indicated a very positive response from the students, including some reflections on their educational journey through medical school. The comments also included several suggestions for developing the activity further. We analyzed these comments by identifying themes and classifying them according to frequency; common (more than three times), repeating (two or three times) and miscellaneous (appearing only once). The themes classification and a transcript of the students’ feedback can be accessed in Supplementary Files 2 & 3.

## Discussion

The most common theme in the feedback was the overwhelmingly positive experience that the students described (79%), which is consistent with other evidence ( Darby, 2007; Doody and Condon, 2012).

‘No other use of debate in MBBS thus far is enjoyable.’

‘It really did, which was very unexpected. I really enjoyed it.’

However, these comments might be influenced by the novelty of debates which Darby (2007) described as an attractive feature. We hoped to reduce this bias by asking the students if they would like more debates in their MBBS curriculum. We hypothesized that students would be less likely to generate more ‘work’ for themselves, and although 70% of the students recommended more debates in their curriculum, this was still a significant majority.

‘Excellent. Having done a prior degree, I was used to my own opinion being valued. In medicine from year one, this is completely shut down.’

‘I have not done any debates since secondary school.’

‘Do more. Once a week?’

Modern medical curricula allow students little room for exploration outside their structure. Our students who had previous exposure to debates had gained this before entering university. 71% of students reported a positive change in their critical thinking and 37% reported a similar impact on their oral presentation skills. These findings are promising, considering that students only had a one-off debate event. However, it is documented that performance and confidence improve with repeating debates ( Dundes, 2001; Hawkins, Fulford and Phan, 2019). We observed that some of the students who reported no improvement suggested that it could be due to the planned lesson being a singular unrepeated event.

‘I think Psychiatry does bring up a lot more ethical issues which are extremely helpful to discuss.’

‘The placement is more influential when considering psychiatry as a speciality.’

The Royal College of Psychiatrists has advocated the use of enriching activities as an essential strategy towards positively influencing medical students’ attitudes towards Psychiatry and increasing recruitment into the speciality ( Royal College of Psychiatrists, 2019). However, we were concerned that the inherent nature of debates highlighting controversies might turn students away from Psychiatry. Hence, we were keen to assess the degree to which educational debates helped or hindered this aim. The students’ comments fit with the evidence that clinical experience is the most influential factor on future career choices in Psychiatry ( Eagles *et al.*, 2007). Nevertheless, using debates as innovative teaching tools can positively influence attitudes towards complex disciplines leading to an increased interest in understanding them ( Malone and Michael, 2018). Only 1 out of 24 reported that the ensuing appreciation for the controversies and dilemmas of Psychiatry would deter them from choosing it.

‘Medical school is arbitrary and about passing exams where there are tick box right answers, so debating both sides of a question does not help this.’

‘It is not well for exams, but there is knowledge value.’

It is known that learners alter their behaviours to achieve the highest possible grade, even bypassing the planned learning experience ( Alinier, 2003; Byrne and Smyth, 2008). Few students expressed a similar process as they were very focused on the assessment, not the learning process. They questioned the value this activity added to their journey towards passing the MBBS exams. However, such a limited view ignores that oral communication, conflict resolutions & critical thinking are integral tools in the inventory of physicians advocating for their patients and even in everyday life (Snider and Schnurer, 2006 p. 9). Debates can challenge this assessment focused culture as the student cannot bypass the learning experience.

‘Love it - do not do near exams.’

Debaters can exert themselves during preparation ( Darby, 2007). We reduced this effort by providing the students with the evidence needed to construct their arguments. Nevertheless, the bulk of the negative comments tackled the timing of the activity. Due to several restrictions in the scheduling process as well as the framework we adopted; the debates event coincided with the end of the placements’ assessments. Hence, the students felt more stressed during the preparation for the debates, and yet the majority still enjoyed it. Going forward, educational debates should be planned at a time that will not compound other exams stress ( Hall, 2011).

‘This was affected the most as I may be able to have an opinion and yet be able to vote for the opposite based on the arguments’

‘Nice to have an environment where everyone listens and accepts you.’

‘Helped analyze previously held beliefs more critically.’

‘I understand how there is no right or wrong answer and psychiatry is not black and white.’

One of the criticisms of debates is that they are inherently binary, which may reinforce dichotomous thinking ( Tumposky, 2004). Contrarily, our students’ comments highlighted an increased perception that Psychiatry is not ‘black and white’ in addition to challenging their personal beliefs. Debates encourage the participants to examine their biases and let go of them, even if temporarily ( Schroeder and Ebert, 1983; Berdine, 1984). They also provide a protected opportunity to entertain less accepted views from the safety of arguing a motion that is removed from the judgement of others ( Shaw, 2012). Musselman (2004) suggested mitigating this problem by introducing a ‘conciliator,’ who directly suggests alternatives to extreme positions. Garrett, Schoener and Hood (1996) mentioned that a post-debate discussion could also be helpful for this purpose. Thus, while the initial arguments of a debate are dichotomous, the ensuing rebuttal allows for a broad spectrum of views.

‘It is a good mixture to promote discussion, but may not benefit everyone.’

Dundes (2001) reported that learners preferred educational debates to lectures. However, Bonwell (1996) found that some learners are more comfortable with the later since it required less involvement. Similar responses echoed in our feedback as few students felt that this activity was not suited for themselves. Thus, we second Hawkins, Fulford and Phan (2019) recommendation for elective participation in educational debates since the effort spent by participants requires some level of motivation. Increasing the allocated marks for this activity in the course would also increase this drive ( Charrois and Appleton, 2013).

‘Vital to do, but may need to be modified for people with anxiety or who hate public speaking.’

‘I understand how it aims to make you consider the content of what someone says as opposed to taking things personally.’

A ‘healthy’ dose of competition and stress may improve the learning experience in debates by guaranteeing preparation ( Schroeder and Ebert, 1983; Saito and Fujinami, 2011). However, the competitive and confrontational nature, not to mention pre-debate and oral presentation anxieties, can hinder socially anxious students and those who are not comfortable with conflict ( Goodwin, 2003, Tumposky, 2004, Hartin *et al.*, 2017). Hence, we designed our framework to reduce pre-debate anxiety & competitiveness. Only 8% of students reported feeling ‘a bit worse’ in their confidence level after the debates. Furthermore, 58% of students reported improved confidence despite 63% of them having no prior debate experience demonstrating that debating with more experienced peers is a confidence-building exercise. Also, 54% of students reported a positive change in their ability to cope with conflict. Thus, affirming that debates develop tolerance of conflict and desensitize the associated anxiety ( Bellon, 2000, Fisher, Lapointe and Peterson, 2001).

Planning the instructional debate experience is vital to make it a productive learning activity ( Hartin *et al.*, 2017). Below, we reflect on our experience as facilitators:

**Faculty Resources:** Setting up the initial structure and material was taxing, which is a frequent deterrent for faculty to consider new learning strategies ( Bonwell, 1996). However, afterwards, our involvement was limited to analyzing students’ feedback, providing pre-debate support and facilitating the event.

**Class Size:** Traditionally, debates are considered small group activity, but they can be adapted for larger audiences ( Combs and Bourne, 1994; Musselman, 2004). To involve all our students, we sometimes had to allocate them into pairs and gave them equal speaking time to guarantee equal participation.

**Debaters Selection:** By giving students the option to choose from multiple motions, we balanced engaging them in a topic they found exciting yet had enough supporting evidence ( Omelicheva, 2006; Malone and Michael, 2018). We allocated them randomly to supporting and opposing arguments to reduce the chance of defending an argument aligned with their personal views which would reduce their gained insights ( Budesheim and Lundquist, 1999). Also, we asked the students for suggestions for future debates.

**Safe Learning Environment:** Debating in front of an audience, naturally, creates social anxiety of being judged against the competing performer ( Omelicheva, 2006). This anxiety and competition are useful to motivate performance but only to a certain level as instructional debates are educational, not competitive activities ( Schroeder and Ebert, 1983; Musselman, 2004). Also, a focus on ‘winning’ communicates a learned behaviour that may negatively impact professional integrity ( Tumposky, 2004). Thus, creating and maintaining a safe learning environment is vital in educational debates ( Garrett, Schoener and Hood, 1996). The strategies below can help towards this aim;

• *Before:* During the introductory session, we provided a clear outline of the event and discussed different debate strategies ( Jackson, 1990; Roy and Macchiette, 2005). We provided both sides with the same evidence for each debate and highlighted that the motions were chosen so that there was no absolute right answer. We affirmed that the activity is both voluntary and formative. We also made it clear that no criticism of each other’s character will be tolerated, thus forming what we called ‘the rules of engagement’. Pre-debate anxiety can be reduced further by giving enough preparation time, reducing the duration of each phase and arranging a mock debate to set expectations ( Candela, Michael and Mitchell, 2003).
• *During:* At the start of the session, we had a short presentation to cement the message of a safe learning environment. Also, we found it helpful to maintain a positive and slightly informal ambience. Having the flexibility between submitting a written versus oral argument can also help participants who find the process overwhelming ( Berdine, 1984).
• *After:* We avoided facilitator or peer assessment, also, after the feedback, we checked in with the group generally and privately with students who appeared to have a negative experience during the debates or requested this support.

**Role of the Facilitator:** Debates work best when the facilitator serves as a scaffold to direct the participants but letting them lead the activity ( Musselman, 2004). Hence, the facilitator’s involvement in the activity was kept minimal, except in three situations:

• Most commonly, we reminded participants of the time boundaries if they got carried away.
• Less frequently, we interfered with maintaining a balance between the supporting and opposing arguments or correct any misinformation ( Shaw, 2012; Hawkins, Fulford and Phan, 2019). We planned this during the closing remark but often skipped it.
• Only once did we have to interrupt the students to reinforce the ‘rules of engagement’. A student criticized the character of the opposing team in their rebuttal but promptly apologized when reminded.

**Assessment:** We opted for self-assessment outcomes as suggested by our focus group and commonly used in literature for assessing debates ( Ang *et al.*, 2019). A summative assessment process might lead to unhealthy competition and anxiety, reducing the educational value of debates ( Hartin *et al.*, 2017). However, several solutions have been suggested;

• Employing a categorical result of ‘Pass’ or ‘Fail’ rather than a numerical score ( Hartin *et al.*, 2017; Hawkins, Fulford and Phan, 2019).
• Assigning most of the grade to pre-debate preparation ( Hawkins, Fulford and Phan, 2019).
• Peer assessment using a rubric marking grid which takes away control from the instructor ( Smith, 1990; Lampkin *et al.*, 2015).

**Online Platforms:** Although not employed here, the classic debate structure lends itself well to the online environment either synchronously via video conferencing ( DeClerk, LaBorde and Smith-Olinde, 2020) or asynchronously via forums ( Lin and Crawford, 2007). This flexibility is a crucial feature considering the challenges posed by the COVID-19 pandemic on traditional teaching strategies.

## Limitations

We can identify four limiting points in our work;

1. Short duration of the activity.
2. Limited employment of research skills.
3. Small sample size.
4. Using of self-assessed outcomes.

We believe that the first two limitations were much-needed adaptations to fit this learning activity into our students teaching program. Moreover, we found that even with these restrictions, the value of using debates in medical education was evident. The small sample size was an arbitrary interference of the COVID-19 pandemic, leading to cancelling all face-to-face clinical placements. Finally, we followed Ang *et al.* (2019) in opting for self-assessment outcomes to reduce participation anxiety. However; we believe that an effective integration into the MBBS curriculum will require applying more objective assessment methods.

## Conclusion

Our students’ feedback and our observations support the evidence of the value of using debates in undergraduate healthcare education. They are ideally suited for preparing learners to be well-rounded healthcare professionals who can advocate for patients and lead change in healthcare policies ( Faust and Paulson, 1998; Hall, 2011). Unfamiliarity with using debates for learning and over-estimating the difficulty of implementation turns away students ( Goodwin, 2003) as well as educators ( Omelicheva, 2006). We hope that this paper will motivate and guide others in adopting and adapting the powerful and ancient but neglected adult learning practice of educational debates.

## Take Home Messages

• Educational debates are collaborative not competitive.
• Educational debates help future clinicians develop their non-clinical skills of communication, critical thinking and coping with conflict.
• Educational debates positively influence medical students’ attitudes towards psychiatry and can help increase recruitment into the specialty.
• Education debates can be flexibly adapted to different fields and platforms.

## Notes On Contributors

**Dr Ahmad Allam** is a core trainee in psychiatry in the NHS in the North-East London Deanery. ORCiD: https://orcid.org/0000-0002-7428-9965

**Mr Ashvin Kuri** is a third-year medical student and the head of the debates club at Barts & The London Medical School. ORCiD: https://orcid.org/0000-0003-0125-4806

**Dr Ali Ajaz** is a forensic consultant psychiatrist in the NHS and a senior clinical lecturer in Psychiatry at Barts and The London School of Medicine and Dentistry.

**Dr Saman Ahmed** is an old age consultant psychiatrist in the NHS and the Dean of Undergraduate Medical Education at North East London Foundation NHS Trust.

**Dr Peter Carter** is a general adult consultant psychiatrist in the NHS and the Director of Medical Education at North East London Foundation NHS Trust.

**Professor Ania Korszun** is a liaison consultant psychiatrist in the NHS and a professor of psychiatry & education at Barts and The London School of Medicine and Dentistry where she is also the lead at the Centre for Psychiatry.
