# Supplementary material for: Critical Psychiatry Debates; A Neglected Tool in Undergraduate Medical Education
Source: MedEdPublish (2016). 2021 Sep 20;10:55. Originally published 2021 Feb 25. [Version 2] doi: 10.15694/mep.2021.000055.2 (PMC10939624; doi:10.15694/mep.2021.000055.2)
Supplement: Peer review history

# Reviewer report: Reviewer response for version 2

**DOI:** 10.21956/mep.20211.r31293

**Published:** 2021-10-01

**Authors:** David Bruce

**Affiliations:** 1 None

This review has been migrated. The reviewer awarded 4 stars out of 5 I had provided a review of the previous version of this paper and again enjoyed reading the authors account of this innovative work. I was struck again by the positive messages that arise from this study -the enjoyment that students had from this experience and their reported increase in their confidence, presentation skills, critical thinking and managing conflict skills. Given the known problems in recruitment and workforce numbers in psychiatry - students reporting a more positive attitude towards Psychiatry and a future career in Psychiatry has to be welcomed. I think the changes improve this paper and my only comment would be that the results from the thematic analysis would be better presented in the results section and not the discussion. This study clearly involved considerable work in setting up and providing space for the debates and I think the helpful tips offered by the authors is excellent. A paper that will be of interest to all in undergraduate education - and many in shortage specialties.

Reviewer Expertise:

NA

No decision status is available

# Reviewer report: Reviewer response for version 2

**DOI:** 10.21956/mep.20211.r31292

**Published:** 2021-09-23

**Authors:** Balakrishnan Nair

**Affiliations:** 1 Centre for Medical Professional Development and University of Newcastle

This review has been migrated. The reviewer awarded 4 stars out of 5 I found this article interesting and innovative. Psychiatry teaching is a vital part in undergraduate curriculum. This format of debating in grey areas in mental health, will stimulate deep learning , critical thinking and motivation. The other benefit is improved communication skills There was good participation rate and the learners were positive in their feedback. As the authors commented , some may find that this innovative teaching interfering with the assessments. The solution may be to make these sessions, as part of the assessment .

Reviewer Expertise:

NA

No decision status is available

# Reviewer report: Reviewer response for version 1

**DOI:** 10.21956/mep.19147.r27687

**Published:** 2021-03-12

**Authors:** Duncan Double

This review has been migrated. The reviewer awarded 2 stars out of 5 Doesn’t seem to be any mention of the content of the debates

Reviewer Expertise:

NA

No decision status is available

# Reviewer report: Reviewer response for version 1

**DOI:** 10.21956/mep.19147.r27688

**Published:** 2021-03-04

**Authors:** David Bruce

**Affiliations:** 1 None

This review has been migrated. The reviewer awarded 3 stars out of 5 In this interesting paper the authors report on the value of structured debates as part of the learning process for undergraduate students during their 4th year Psychiatry placement. They describe the use of debate to improve communication skills and critical thinking in students, and piloted and evaluated a 2 hour debating session based on 6 motions that meet ideal debate criteria. 24 students out of a cohort of 26 participated. The evaluation was an 11 item questionnaire with free text comments. The authors also provide their reflections on their experiences as educators and facilitators and provide helpful practical advice. The results are positive with students enjoying the experience and reported an increase in their confidence, presentation skills, critical thinking, managing conflict skills and in addition having a more positive attitude towards Psychiatry and a future career in Psychiatry. The paper is well written and easy to read and provides the questionnaire and free text comments and analysis as supplementary files. As a reader I would have like to see details of the six motions that were used in the debates also included. The paper reports the descriptive statistics from 7 of the 11 questions presented in table form, and data from 2 of the other questions. The order of data in the table is different to the sequence of the original questionnaire and 2 questions are commented on and the final question about future topics for debates is not provided. This slightly confusing (but perfectly logical) and could perhaps be explained in the paper. Not all readers might download the supplementary files – and I wondered if there is a way to present the free text comments and analysis in the actual paper. The free text comments for each question are dealt with by thematic analysis – which might better appear in the results section. I felt that this part of the paper was quite complex and as thematic analysis had been completed then more detail of how this was carried out and quality assured should have been provided. There were a large number of themes (31 for 100 comments) and while comments were matched to a theme – there seemed like a risk of getting comments out of their context. An example would be using the comment “I have not done any debates since secondary school” to discuss the wish for more debates – when this comment was in response to the question about previous debating experience. Perhaps the free text comments might have been used to further illustrate the results seen in each question.The authors experiences and recommendations for others wishing to set up such debates was very helpful – and perhaps should have been included in the abstract. As a minor point, I was not clear about what was meant by a limit to the study being limited employment of research skills. I think this paper will be of interest to all involved in undergraduate teaching and is a positive and helpful addition to teaching methods.

Reviewer Expertise:

NA

No decision status is available

# Reviewer report: Reviewer response for version 1

**DOI:** 10.21956/mep.19147.r27686

**Published:** 2021-03-02

**Authors:** Balakrishnan Nair

**Affiliations:** 1 Centre for Medical Professional Development and University of Newcastle

This review has been migrated. The reviewer awarded 4 stars out of 5 I find this article interesting and innovative. We need to change the attitude of students towards psychiatry by making them active learners. Whilst doing this , the project also improved their confidence, communication , and critical thinking skills . It would have been interesting to see the topics for these debates .

Reviewer Expertise:

NA

No decision status is available
